# Childhood behavioral inhibition is associated with impaired mentalizing in adolescence

**DOI:** 10.1371/journal.pone.0195303

**Published:** 2018-03-29

**Authors:** Sergi Ballespí, Ariadna Pérez-Domingo, Jaume Vives, Carla Sharp, Neus Barrantes-Vidal

**Affiliations:** 1 Departament de Psicologia Clínica i de la Salut, Universitat Autònoma de Barcelona, Barcelona, Catalunya, Spain; 2 Department of Mental Health, Fundació Sanitària Sant Pere Claver, Barcelona, Catalunya, Spain; 3 Institut Docent, Fundació Sanitària Sant Pere Claver, Barcelona, Catalunya, Spain; 4 Departament de Psicobiologia i de Metodologia de les Ciències de la Salut Universitat Autònoma de Barcelona, Barcelona, Catalunya, Spain; 5 Developmental Psychopathology Lab, University of Houston, Houston, Texas, United States of America; 6 Centre for Biomedical Research Network on Mental Health (CIBERSAM), Instituto de Salud Carlos III, Madrid, Spain; University Hospital Heidelberg, GERMANY

## Abstract

Recent advances suggest that impairment in social cognition (SC) may play a role in the development of social anxiety (SA). However, very few studies have analyzed whether SA fosters poorer social-cognitive development as it leads to social avoidance. This study aimed to analyze whether retrospectively assessed behavioral inhibition (BI) (i.e., an early form of SA) in childhood is associated with a deficit in social cognition operationalized as impairment of mentalizing (MZ) in adolescence. A sample of 256 adolescents (range: 12–18 years; mean age: 14.7 years; *SD* = 1.7) from general population were assessed for MZ capacities and retrospective BI through self-report and interview measures. Results comparing three groups of adolescents with different levels of childhood BI (low, moderate or high) and controlling for concurrent SA and depression reveal that the higher the level of BI, the lower the level of MZ. These results were consistent for almost all mentalization measures, including when both extreme (i.e., high vs. low BI) and non-extreme (i.e., high vs. moderate BI) were compared in both self-report and interview measures and in both dimensions of MZ (i.e., MZ referred to others’ and to own mental states). These findings support that childhood forms of SA are associated to deficit in SC in adolescence. A possible bi-directional relationship between SA and SC, and the role that it may play in the pathway to clinical SA are discussed.

## Introduction

Social cognition refers to the perception, interpretation, and processing of all information relating to a person’s social environment and relationships [1]. The term social cognition is interchangeable in the literature with other terms (e.g., Theory of Mind, Mentalization). In fact, the historical interest in how humans are aware of one’s own and others’ mental states is patent in the number of approaches that its study has produced. For instance, constructs such as *social intelligence* [2], *metacognition* [3], *Theory of Mind (ToM)* [4], *mind-blindness* [5], *inter and intra-personal intelligences* [6], *emotional intelligence* [7] and others (e.g., *mind-reading* or *insight*) seem to be referred to the same Higher-Order Cognition [8]. In this context, the recent Mentalization paradigm contributes to systematize a field with multiple historical approaches.

Mentalization or mentalizing (MZ) emerges from recent advances in several disciplines (developmental psychology, neuropsychoanalysis, social neuroscience) to address a well-studied social-cognitive capacity from a new multidimensional point of view [9,10]. Thus, MZ paradigm organizes the ability to recognize intentional mental states (i.e., not only thoughts, but also feelings, emotions, desires, impulses) [11] according to four basic dimensions [12]. MZ is a multidimensional construct that refers both to self and others’ mental states, both to cognitive and emotional functioning, both to implicit-automatic and explicit-controlled activation of the ability, and it can be based on internal versus external features of self and others [13,14]. Given that MZ constitutes a new broad paradigm that integrates several constructs and systematizes both self-awareness phenomena (e.g., insight, mindfulness, metacognition), and the wide field of social cognition (e.g., empathy, Theory of Mind (ToM), mind-reading), this is the perspective adopted in the present study in order to assess two dimensions of this capacity: MZ referred to one’s own and to others’ mental states.

The awareness of one’s own and others’ mental states not only allows the understanding and prediction of human behavior, but it is related to good social functioning and mental health [15–17]. By contrast, evidence supports that MZ impairment is related to several psychopathological conditions [18]. For instance, a diminished capacity to imagine others’ mental states (i.e., ToM deficit) is associated to autism spectrum disorders [19–21] and psychosis [17,19,20,22,23]. Deficiencies in complex mental state reasoning and in simple mindreading have been found, respectively, in adolescents and children with psychopathic symptoms [24–28], and the tendency to attribute excessive or unrealistic mental states has been described in borderline personality disorder [12,29–32]. In addition, recent studies showed that problems in MZ are also present in depression [33], anxiety [34,35], trauma-related disorders [36], eating disorders [37], attachment-related problems [11,38] and addictions [39,40]. Thus, problems in MZ capacity are present in several psychopathological conditions. Given the possibility that social cognition may play an important role in the presence of psychopathology, there is growing interest to extend the study of this Higher Order Cognition [8] to other psychopathological domains.

Social cognition is especially interesting to be analyzed in disorders with an important social component. In the Social Anxiety Spectrum [41] the role of cognition (i.e., attention, perception and memory biases) [42,43] and meta-cognition [44] have been widely documented, but the specific role of *social* cognition remains relatively underexplored [35,45]. This is important because, in contrast to other anxiety phenomena, the basic fear in social anxiety (SA) is precisely “social” (i.e., SA is defined by the fear of scrutiny, social interaction, and negative judgment of others [46].

Interestingly, as Banerjee and Henderson [45] already pointed out, the relationship between social anxiety and social cognition is very probably bidirectional, and this means that they may influence each other along development. Thus, in addition to the contribution of social cognition to social anxiety, it might be reasonable to analyze how social anxiety may contribute to the development of social cognition.

Despite this, among the relatively small number of studies analyzing social cognition in SA, most of them are focused on how social cognition contributes to SA [47–54], but very few of them analyze how SA impacts social cognition development. This is important because if SA and social cognition feed each other in some way, maybe clinical levels of SA in adolescence or adulthood depend to some extent upon this interaction.

Most of the studies about social cognition in SA are based on adult samples with clinical levels of SA [47–52]. In contrast, there are relatively few studies focused on how non-clinical forms of SA in early stages of development [i.e., temperamental shyness or behavioral inhibition (BI); [46,55–57] impact the development of social cognition.

In this matter, the preliminary investigation of Banerjee and Henderson [45] highlighted the interplay between social cognition and SA and established that socially anxious children, particularly those with high levels of shy negative affect, experienced specific social-cognitive difficulties in understanding the links between emotions, intentions, and beliefs in social situations. According to this pioneering study, any condition that leads to the avoidance of social interaction may affect the “practice” and development of social cognition. Therefore, an important question is whether SA along development, which is typically associated to social avoidance, may affect social cognition or, in other words, MZ capacity.

After the correlational study of Banerjee and Henderson [45], two lines of findings report variations in MZ as a consequence of early forms of SA. According to the emotional reactivity hypothesis [58] suggesting that emotional arousal impairs ToM development, there is evidence about an inverse relationship between early SA and social cognition. For instance, Suway, Degan, Sussman, and Fox [59] found that children with high levels of BI at 24 months of age showed less ToM understanding at 36 months, in comparison with non-behaviorally inhibited children, supporting the notion of a possible impairing effect of BI on MZ capacities. This study is consistent with previous ones supporting that the higher the SA (i.e., shyness, BI), the lower is level of social cognition [60,61].

By contrast, there is another line of results pointing that early forms of SA may lead children to an observant position regarding social phenomena that may help to improve social cognition development. In this sense, Mink, Henning, and Aschersleben [62] found that shy temperament at 18 months was associated with more sophisticated ToM abilities at age 3. According to previous studies [63,64], in this case, these authors interpreted that a more observant temperament (shyness) provided better opportunities for reasoning about others’ mental states.

Finally, some studies like those of Colonnesi and colleagues [65] and La Bounty and colleagues [66] report findings partially supporting both lines of results. Thus, it is still not well-known whether shyness might benefit or impair social cognition development. Both findings provide evidence for an influence of early SA in MZ abilities, but maybe the differences in measures of shy temperament and ToM, differences on how ‘shy’ or ‘inhibited’ condition is defined, as well as differences in age sample and time of follow-up may explain discrepant results.

Most of the studies analyzing this topic are focused on an early and narrow period of development usually placed [59,62,63,65,66] or beginning at the preschool age [60]. However, although there is evidence about errors of metaperception in socially anxious adolescents [67], there is no study analyzing whether early forms of SA in childhood (such as BI) predict MZ capacity in adolescence.

Therefore, the first aim of the current study was to analyze if BI in childhood may be associated to MZ in a later period of development. Specifically, we aim to analyze whether MZ in adolescence is associated to BI in childhood, retrospectively assessed. In contrast to some previous findings, we assume that the tendency to avoid social interaction over years, due to a high level of temperamental BI, may impede practice and sophistication of MZ abilities and therefore lead to lower levels of MZ in adolescence. Thus, we predict that a group of adolescents with high level of childhood BI, retrospectively assessed, would show lower MZ abilities than a group with no BI.

However, considering a possible explanation to previous discrepant results, we also hypothesized that, although a high level of BI may impede MZ development—because anxiety leads to social avoidance and excessive arousal blocks mental functioning [68] it is possible that moderate levels of BI may be associated with higher mentalizing capacity because BI may foster a tendency to pause and observe rather than immediate action. This combined hypothesis may explain why some studies find that early SA leads to better ToM, and may help to integrate the previous conflicting results.

Thus, the second aim of this study is to examine whether a group of adolescents with a moderate level of BI in childhood show higher levels of MZ in adolescence than a group with high childhood BI, and even than a non-inhibited group.

In addition, given that most preceding studies have given preference to analyzing the relationship between SA and MZ of others’ mental states, the third aim of the current study was to see whether early SA, operationalized here as a measure of BI, not only affects social cognition but also affects the ability to consider one’s own mental states. Neuroscience research reveals that MZ capacities referred to self and others’ share several neurological routes [69], and other evidence suggests that the impairment of emotional knowledge in SA may be more pronounced in the intrapersonal (i.e., own emotions) than in the interpersonal form (i.e., others’ feelings) [70]. Therefore, we predicted that the group of adolescents with high BI in childhood would show MZ problems both referred to self and others’ mental states.

In light of preceding findings [71] suggesting higher MZ capacity in girls compared to boys [72,73], and evidence of higher prevalence of SA in girls than in boys [74], all the analyses were controlled for sex. Given the wide range of age considered (i.e., 12 to 18 years old), the effect of age also taken into account. In addition, given that 1) anxiety is usually considered a backstage of depression [75,76], 2) there is specific evidence for secondary depression associated to SA [77–80], and the well-known association between MZ problems and depression [33,75,76], depressive symptoms were controlled for in all analyses in order to avoid the confusion effect of depression. For the same reason, given that BI usually leads to SA in adolescence [55], SA in adolescence was also controlled for in order to avoid the confusion of childhood BI and adolescent SA effects on MZ capacities, and thus obtaining a more accurate measure of the relationship between childhood BI and adolescent MZ.

## Materials and methods

### Ethics statement

The study meets ethical standards according to Declaration of Helsinki and it has been approved by the Ethics Committee of the Universitat Autònoma de Barcelona (CEEAH) (Spain). (Num. CEEAH: 2603). All families provided written informed consent.

### Participants

Participants were recruited in the second phase of a comprehensive project on SA. This project was designed with two phases in order to reduce the cost of the field work. It started with a sifting phase aimed to encourage high participation in order to identify a large number of participants with high SA. A second phase was designed to carry out further assessments with a smaller sample with over-representation of SA.

The resulting sample consisted of 260 participants. From those, 256 (137 girls, 53.5%), aged 12–18 years old (*M* = 14.7, *SD* = 1.7), provided valid data (i.e., 98.5% of the initial 260); 70.7% came from families with middle socio-economic level (11.6% low; 17.7% high) and approximately 87% were Caucasian (White-European), 9% Arabic, 2% Asian, and 2% Latino.

### Measures

#### Mentalization

The **Mentalization Questionnaire (MZQ)** [81] is an instrument designed to quantify MZ impairment in clinical populations. It consists of 15 items scored from 1 to 5 based on the level of agreement with every statement. Hausberg et al. [81] reported satisfactory internal consistency (Cronbach’s *α* = .81), and test-retest reliability (*r* = .76), as well as sufficient evidence for validity of the original version. The Spanish version of the MZQ [82] shows moderate internal consistency (Cronbach’s *α* = .79), moderate test-retest reliability (ICC = .65), and evidence for validity based on correlations with measures of psychopathology (*r* ranged from .32 to .55) and an inverse relationship with measures of secure attachment (*r* = -.32). The internal consistency of the MZQ in the current sample is *α* = .77.

The **Brief Reflective Function scale (BRF)** [83] is a 4-item instrument inspired in very short scales such as the Relationship Questionnaire (RQ) [84]. As the RQ, the BRF provides 4 global descriptions (e.g., *Item 1* - *“I am a person who always knows how I am feeling; I am in touch with my feelings*, *and I even know why I feel the way I do”*) that can be scored from 1 (*Totally disagree*) to 7 (*Totally agree*). The first item is referred to own mental states, while items 2 to 4 are referred to others’ mental states (e.g., *Item 4 –“I am usually able to read others’ minds*, *I understand what happens inside other people*, *and I can “see” their intentions*, *desires or feelings”*). The BRF is composed of one dimension that explains 54% of the total variance, and shows moderate internal consistency (Cronbach’s *α* = .70), sufficient test-retest reliability (ICC ranging from .47 to .62 for the 4 items), and evidence for the association with several measures of related constructs (ranging from .20 to .45). The subscale referred to others’ mental states (items 2 to 4) shows an internal consistency of *α* = .76. The internal consistency of the BRF in the current sample is *α* = .73 for the whole scale and *α* = .80 for the others’ subscale.

The **Adolescent Mentalizing Interview (AMI)** [85] is specifically designed for measuring MZ in adolescence. It consists of two guided exercises structured in 7 questions, which can be scored from 0 (*No mentalizing*) to 4 (*Sophisticated mentalizing*). The first exercise asks the adolescent about the dynamics of mental states based on the interaction of the 3 characters of a picture story. The second one asks about the mental states of the participant in relation to two Very Close Others (VCO; e.g., parents, boy-/girl-friend, best friends) previously selected by the participant. The factor analysis of the AMI showed one global dimension explaining 64% of the total variance and an excellent internal consistency (Cronbach’s *α* = .90). Varimax rotation revealed a 2-factor structure with good internal consistency both for the items of the first part (i.e., *Story*: MZ referred to others’ mental states; *α* = .85) and the second one (i.e., *VCO*: MZ referred to self; *α* = .86). Correlations of AMI with measures of the same construct (from .21 to .47) provide evidence for construct validity. Correlations between independent interviewers ranging from .79 to .88 for the items (ICC = .91 for the total score) support inter-rater reliability.

The **Trait Meta-Mood Scale (TMMS)** [86] is a self-report measure designed to assess individuals’ beliefs about their own emotional abilities (i.e., identification, understanding and regulation). The TMMS assesses MZ referred to own mental states. Specifically, it consists of 24 items (e.g., ‘I can’t make sense out of my feelings’) referred to 3 different aspects of meta-cognition (i.e., attention, recognition, comprehension) for which participants are required to rate the extent to which they agree on a 5-point scale (from 1-*Strongly disagree* to 5-*Strongly agree*). The TMMS shows moderate internal consistency (Cronbach’s *α* between .82 and .87) and good convergent and discriminant validity. The Spanish version [87] shows moderate to good internal consistency (Cronbach’s *α* from .86 to .90) and acceptable test-retest reliability (*r* comprised between .60 and .83). The internal consistency in the current sample is excellent (Cronbach’s *α* = .90).

### Temperament and psychopathology

The **Behavioral Inhibition Scale (BIS)** [88] is structured in two parts. The current study is based on the second part of the BIS, which is used to retrospectively assess BI in childhood from the adolescents’ point of view. The first part of the BIS consists of 4 items with which parents and children rate the degree of children’s behavioral inhibition on 4-point Likert scales with 1 = *never*, 2 = *sometimes*, 3 = *often*, and 4 = *always*. Cronbach’s *α* ranged between .88 and .95, and test-retest correlation of .77 support good reliability for the original version. Similar results (Cronbach’s *α* = .78, test-retest ICC = .67) were obtained for the Spanish version [89]. Authors refer moderate correlations with the scales of the Early Adolescent Temperament Questionnaire–Revised which suggest good convergent and discriminant validity. The second part presents three descriptions (e.g.: *“As long as I remember*, *I am shy when I have to talk to an unfamiliar person…”*) and asks the informant which one better identifies him or her BI profile (inhibited, moderate, non-inhibited) from a developmental point of view.

The **Beck Depression Inventory 2 (BDI-2)** [90] consists of 21 self-evaluative items, each with three symptom-choices reflecting the respondent’s experience over the course of a week. The Spanish adaptation [91] shows good psychometric properties (e.g., Cronbach’s *α* = .87). The internal consistency in the current sample is excellent (Cronbach’s *α* = .90).

The **Social Anxiety Scale for Adolescents (SAS-A)** [92] contains 18 self-statements referred to SA (e.g., “I worry about what others think of me”) and 4 filler items. All of them are rated on a 5-point Likert scale according to how much the item “is true for you”, ranging from 1 (not at all) to 5 (all the time). The Spanish adaptation [93] shows adequate internal consistency (Cronbach’s *α* between .76 and .91), good test-retest reliability (*r* between .75 and .86) in a 10-days interval, and evidence for convergent validity. The internal consistence of SAS-A in the current study was excellent (Cronbach’s *α* = .90).

### Procedure

Families were informed about the study through a letter widespread by the school, and they were also invited to a meeting to solve any doubts regarding the study. After obtaining the informed consent, data were recruited in the schools. Missing data and response-mistakes were detected and solved through a new contact with participants. In order to analyze whether different levels of BI, retrospectively assessed, may be associated to variations in MZ, groups of high, moderate and low childhood BI are defined on the basis of the second part of the Behavioral Inhibition Scale.

### Statistical analysis

Multiple lineal regressions were performed to test the effect of different degrees of BI in childhood (high, moderate, low) on different measures of MZ in adolescence, controlling for sex, age, depressive symptoms, and SA in adolescence.

Regression backward model selection was conducted, using IBM SPSS Statistics v20.0 package [94] to fit each model. Assumptions of linearity, independence, homoscedasticity and normality were checked, as well as the presence of influential values. The results of the association between BI and each MZ measure are presented as linear regression coefficients (B) for quantitative responses, reporting 95% confidence intervals (95% CI), and P-values (P).

## Results

### Comparing adolescent MZ capacities between groups with high and low childhood BI

Table 1 shows descriptive statistics of MZ measures for every group of BI. These groups are based on the level of BI in childhood, retrospectively assessed with the second part of the Behavioral Inhibition Scale. On the base of the participants’ responses, the group with high BI includes 37 of the 256 participants of the whole sample (15%), the group of low BI includes 80 (31%) and the moderate group includes the remaining 138 participants (54%).

**Table 1:** Descriptive statistics of dependent variables by group of behavioral inhibition (BI).

			High BI (n = 37) *M* (SD)		Moderate BI (n = 138) *M* (SD)		Low BI (n = 80) *M* (SD)
***Global measures of MZ***							
	Self-report	MZQ	46.8 (10.7)		44.7 (9.1)		41.7 (8.5)
		BRF	13.3 (4.4)		15.2 (4.2)		16.9 (4.0)
	Interview	AMI	12.0 (4.8)		14.6 (4.6)		15.4 (5.1)
***MZ referred to one’s own MS***							
	Self-Report	BRF-self	4.5 (1.7)		4.9 (1.3)		5.4 (1.4)
		TMMS	69.0 (14.1)		72.2 (14.3)		79.4 (16.0)
	Interview	AMI-part B	6.3 (3.1)		7.9 (2.9)		8.3 (3.4)
***MZ referred to others’ MS***							
	Self-Report	BRF-others	12.8 (4.0)		14.3 (3.6)		15.4 (3.3)
	Interview	AMI-part A	5.7 (2.1)		6.7 (2.0)		7.1 (2.0)

Notes: AMI = Adolescent Mentalizing Interview (range 28); AMI-A (range 12); AMI-B (range 16). BI = Behavioral Inhibition. BRF = Brief Reflective Function scale (range 24); BRF-self (range 6); BRF-others (range 18). MS = Mental states. MZ = Mentalizing. MZQ = Mentalizing Questionnaire (range 60).

Table 2 shows the effect (*B*), the 95% confidence interval (95% CI) of *B* and the *p* value, of BI (High vs Low, High vs Moderate, Moderate vs Low) on different MZ measures. The first column of Table 2 shows the effects of having high BI vs. low BI on MZ. Adolescents with high BI in childhood show significantly lower MZ capacity, in almost all measures, compared to adolescents with low BI in childhood. Thus, for global MZ measures (i.e., encompassing both MZ referred to own and others’ mental states), the group with high BI in childhood scored between 2.2 and 5.3 points lower than the low BI group for the BRF (i.e., 10% to 20% lower, given the range of BRF: 0–24), and between 1.8 and 5.4 points lower (i.e., 5–20% lower) in the AMI interview. This result is consistent across global and specific MZ measures, both for MZ referred to own and others’ mental states and both for the self-report and interview. Thus, with the only exception of the measure of MZ deficit provided by the MZQ, these results suggest that a high level of BI in childhood is associated to impaired MZ capacities in adolescence when compared to low level of BI.

**Table 2:** Multiple lineal regressions comparing levels of mentalizing among groups with different levels of BI.

		High BI vs. Low BI		High BI vs. Moderate BI		Moderate BI vs. Low BI	
		B [95% CI]	*p*	B [95% CI]	*p*	B [95% CI]	*p*
***Global measures of MZ***							
Self-report	MZQ	-.96 [-4.21, 2.28]	.561	-1.57 [-4.43, 1.29]	.281	.61[-1.45, 2.67]	.560
	BRF	-3.76 [-5.34, -2.18]	.000	-2.00 [-3.47, -.53]	.008	-1.76 [-2.84, -.68]	.001
Interview	AMI	-3.57 [-5.38, -1.77]	.000	-2.38 [-4.06, -.70]	.006	-1.20 [-2.49, .10]	.070
***MZ referred to one’s own MS***							
Self-Report	BRF-self	-.96 [-1.47, -.46]	.000	-.50 [-.97, -.03]	.035	-.46 [-.81, -.11]	.009
	TMMS	-10.07 [-15.94, -4.21]	.001	-3.12 [-8.59, 2.34]	.263	-6.95 [-10.91, -2.99]	.001
Interview	AMI-part B	-2.13 [-3.30, -.95]	.000	-1.49 [-2.58, -.39]	.008	-. 64 [-1.48, .20]	.135
***MZ referred to others’ MS***							
Self-Report	BRF-others	-2.37 [-3.47, -1.27]	.000	-1.22 [-2.27, -.18]	.021	-1.15 [-1.83, -.46]	.001
Interview	AMI-part A	-1.37 [-2.15, -.59]	.001	-.97 [-1.70, -.24]	.009	-.41 [-.96, .15]	.151

Notes: AMI = Adolescent Mentalizing Interview (range 28); AMI-A (range 12); AMI-B (range 16). BI = Behavioral Inhibition. BRF = Brief Reflective Function scale (range 24); BRF-self (range 6); BRF-others (range 18). MS = Mental states. MZ = Mentalizing. MZQ = Mentalizing Questionnaire (range 60). All the analyses were carried out adjusting for sex, age, and the level of SA and depressive symptoms in adolescence.

### Analyzing if moderate childhood BI is associated to higher adolescent MZ than high and low childhood BI

The second and third columns of Table 2 reflect regressions comparing a group with a moderate level of BI with groups of high and low BI, respectively. The second column shows that to be highly inhibited implies lower MZ capacities even in comparison to a moderate group of BI, again for almost all measures, except for the MZ deficit measure (MZQ) and the specific measure of meta-cognition (TMMS).

Finally, the third column indicated that all significant differences were against predictions, that is, participants with intermediate levels of BI presented lower MZ capacities compared to those with low BI. In this case, though, significant differences only emerged for self-report measures.

## Discussion

The first aim of the current study was to analyze whether retrospectively reported BI in childhood, which is considered an early form of SA [41,55], is associated with impaired MZ capacity in adolescence. Retrospective BI was evaluated with the second part of the Behavioral Inhibition Questionnaire (BIS), which consists of presenting three descriptions and ask the informant which one better identifies his or her profile. The resulting groups, based on the responses of the participants to the BIS—i.e., not based on cut-offs selected by the researchers—suit relatively well the criteria of the group of Harvard [95–97], which establishes that 15% of the general population can be considered qualitatively ‘inhibited’. However, this percentage was established by previous studies of general populations [96,98,99], while the current sample shows an overrepresentation of SA. This suggests that the retrospective self-classification of historically ‘inhibited’ might be underestimated. In fact, to the well-known biases attributed to retrospective measures [100], it must be considered that also variations in MZ, especially in self-referred dimensions (i.e., the capacity to be aware of one’s own mental states or meta-cognition) can affect any self-report.

In any case, the over-representation of SA in the current sample was a decision design of the comprehensive project to make easier the study of SA phenomena. So it should also benefit the current study. Thus, the underestimation of historically ‘inhibited’ children in the current sample suggests that some group differences might be attenuated since some ‘inhibited’ children have been wrongly self-classified in a non-high inhibition group.

Having said that, the analysis of current results supports that having been highly inhibited in childhood is indeed associated to lower MZ capacities in adolescence, both compared to those with low BI and even to those with intermediate BI along development. This is important because the fact that also contiguous groups (intermediate vs. high BI), show differences in MZ capacities reinforces the implications derived from the comparison of extreme groups (i.e., high vs. low BI).

Moreover, results do not only support that high BI in childhood is associated to poorer social cognition in adolescence, but also indicate that inhibited children exhibit impairments in both self- and other- dimensions of MZ. This is reasonable since social interaction seems necessary both to develop social cognition and also meta-cognition, since others’ point of view (i.e., *mirroring*; [101]) may help to increase self-knowledge in terms of recognition and management of own mental states in the social world [102].

To our knowledge, a relatively small number of studies analyzed how SA may affect social cognition, and their results go in two opposite directions. While some studies provide evidence for Hare’s [58] emotional reactivity hypothesis, which supports that less reactive temperaments lead to more sophisticated social-cognitive skills [59–61], other studies clearly support the opposite, that is: that shy / inhibited temperaments lead to observant attitudes that foster social-cognitive development [62–64]. These conflicting results lead Lane and colleague’s [64] to point the necessity of carefully review Hare’s hypothesis in the case of humans, and this was what motivated current research to consider 3 different levels of childhood BI here.

According to previous studies [59–61], the current results can be interpreted in light of what classic research on BI supports [95–99, 103]: Inhibited children are more socially reticent [104] and less likely to approach or engage others in social interaction [105]. As a consequence, they have fewer opportunities to learn the abilities necessary for competent social cognition [106]. In turn, impoverished social cognition may contribute to further declines in social understanding and therefore more social avoidance [107], thus suggesting that the relationship between SA and social cognition may be bi-directional. This apparent reciprocal influence, already suggested by previous research [45], may be an important point to explain the stability of both SA and social cognition deficit, and allows to combine the current findings with those supporting that problems in MZ may play a role in the development of SA (e.g., [47–52]).

By contrast, the current results do not support those of previous studies reporting better MZ capacities in preschoolers with higher shyness [62–64]. It is worth noting that it was precisely the existence of conflicting results what suggested us that maybe they were different levels of SA that may influence the development of SA differently. Therefore, it was hypothesized that, although a high level of BI could be so arousing to lead to social avoidance and consequently to impair social cognition, maybe an intermediate level of SA may lead children to stop and observe—instead of avoid or flee—thus improving social cognition instead of impairing it. This may go along with previous studies supporting Lane and colleagues’ statement [64], and it is possible to apply Wellman and colleague’s postulate to *moderate* shyness, considering that moderate levels would allow for a more quietly observant stance toward social interaction, which would in turn yield to better insight (i.e., MZ) of interpersonal processes. However, against predictions, current results are clear in refuting this hypothesis, and indeed support that levels of BI are proportional to the level of impairment in MZ ability.

Interestingly, differences between the intermediate and the low BI groups only appear regarding self-reports and not for interview measures. In this context, it is interesting to note that an interview, which is an emotional-arousing situation for people with SA, did not detect differences in MZ abilities between groups with non-high SA (i.e., low versus moderate BI), while self-reports, reflecting informants’ appraisals of their capacity to MZ, indeed supported lower MZ abilities in people with history of intermediate BI. Although participants with low and moderate childhood BI show no differences in MZ functioning over an interview, it seems as if a history of intermediate BI may influence self-perception of MZ capacity. This is important because one of the expected limitations of this study is precisely the use of self-reports to assess MZ—because an impaired MZ capacity, especially regarding one’s own mental states, may affect all psychological assessments. Thus, it could be expected to find differences with the interview—considered more reliable—and not with self-reports, but not the opposite. However, current results indicate, in light of the interview measurements of MZ, that to have been inhibited is associated to poorer MZ capacity compared to moderate and low childhood BI, but there are no differences between having been moderate- and non-inhibited. This sheds light on an additional issue: the potential differences between MZ measures heighten the debate about the still unsolved difficulty to apprehend this Higher Order Cognition [108,109]. We tried to attenuate this problem using different measures of MZ in the current study—i.e., a multimethod perspective—, but this also obligates researchers to carefully consider the conclusions derived from the current findings until appropriate replication with improved measurement can be carried out.

A final prediction of this study concerned the multidimensionality of the impairment of MZ associated to SA. In light of previous research [35,44] and classical theories about SA [46,55–57], it was predicted that developmental BI may be associated not only with differences in social cognition (i.e., MZ referred to others’ MS), but also to metacognition (i.e., MZ referred to own MS). Results supported this hypothesis with both self-report and interview measures, both when the high BI group is compared to low BI and also to moderate BI group. This finding seems to be robust given the dose-response pattern observed for the influence of SA on MZ abilities.

However, there are two exceptions to this conclusion. The first one is the measure of MZ deficit provided by the MZQ. This is interesting because the MZQ is a specific measure of *deficit* and this is the only measure in which any difference was found. The MZQ was used because it was the only evidence-based psychometric measure of MZ available at the time when the study was conducted. However, precisely for being an instrument specifically designed to detect deficit (i.e., clinical impairment) in MZ in adults from clinical population, it is possible that its items (e.g., *“Sometimes feelings are dangerous for me”*, *“Most of the time is better not to feel anything”*) are not the most suitable to capture differences in a sample of adolescents from general population.

The second exception implies that adolescents with high and moderate BI along childhood do not differ in the meta-cognition specific measure (i.e., TMMS-24), although they do in the other two measures referred to the self-MZ dimension. The TMMS-24 is a general measure of meta-cognition with items referred to attention, recognition and comprehension of own mental states. In contrast to the more precise self-MZ measures (i.e., BRF, interview), in this “G-factor” of meta-cognition, results indicate that the moderate group is clearly nearer to the high BI group than to the non-inhibited group. This reinforces the idea that SA along development (either high or moderate) not only affects social cognition but also metacognition, and that on this regard there is no difference across levels of SA, because differences appear on the basis of presence (either high or moderate) compared to absence of SA (i.e., the low BI group).

In conclusion, this study indicates that retrospective assessments of childhood BI are associated to poorer MZ in adolescence, and that not only the social cognition dimension is impaired (i.e., MZ referred to others’ mental states), but also meta-cognition. Contrary to our predictions, intermediate levels of BI are not associated to an increased MZ capacity in adolescence. By contrast, they are associated to and intermediate impoverishment of MZ. These findings underscore the relevance of taking into account at early developmental stages the potential implications of BI in terms of a potential detrimental effect for MZ abilities beyond its well-known impact on increasing risk for SA.

### Strengths and limitations

This study has some limitations. First, the self-selected sample, based on voluntary participation, does not guarantee the generalizability of the results. In addition, although the over-representation of SA in the sample might facilitate the study of SA phenomena, it is also detrimental to representativity. Third, developmental BI has been established on a retrospective self-report measure. In this sense, it must be wondered whether possible MZ variations may affect self-reports. This can be important because in order to make possible the assessment of a big number of participants, apart from the interview, most measures came from adolescent self-reports. A multiinformant assessment procedure could be used here to diminish this shortcoming, but two reasons lead to reject this idea. On the one hand, there is the difficulty to find cost-effective methods to assess the multidimensionality of MZ. On the other hand, it was dismissed to obtain parents and teachers measures on several constructs to avoid increasing the complexity derived from potential discrepancies among informants. To address these limitations, further research should test the current hypotheses from a longitudinal perspective using more sophisticated measures of BI and MZ.

The study has also several strengths. Despite the retrospective design, this is the first study to explore the relationship between BI in childhood and MZ in adolescence. It is also the first in considering three levels of BI (i.e., low, moderate, high) instead of comparing only two extreme groups, which allows for examining dose-response associations. Also, it takes into account several measures of MZ from a multi-method assessment perspective for measuring two dimensions of MZ in the same sample (i.e., self and others). Finally, the use of a broad sample from a non-clinical population allowed to cover a wide range of non-clinical SA, and also made possible the statistical control of several potential confusion variables such as age, sex, the adolescent concurrent level of SA and depression.

## Supporting information

S1 File SPSS data file. (ZIP)
